# Peer Review of “The Influence of COVID-19 Vaccination on Daily Cases, Hospitalization, and Death Rate in Tennessee, United States: Case Study”

**DOI:** 10.2196/32462

**Published:** 2021-08-12

**Keywords:** COVID-19, pandemic, vaccination, vaccine, strategy, vaccination strategy, hospitalization, mortality rates, older adults, mortality

*This is a peer-review report submitted for the paper “The Influence of COVID-19 Vaccination on Daily Cases, Hospitalization, and Death Rate in Tennessee, United States: Case Study.”*

## Round 1 Review

### General Comments

This paper [1] demonstrates that vaccination affects different age groups during the third wave of COVID-19. It shows that vaccination changed transmission rates and resulted in a reduction in hospitalization and death rate.

### Specific Comments

This paper has used exciting data during a challenging time to help other regions that are far behind the United States to set their policies and see how prioritizing older people changes statistics. So, I think this paper adds significant points using great data (around 3 months) to contribute to the COVID-19 literature.

#### Major Comments

1. In the Introduction, it is essential to use other studies to compare different states or countries and other related research. Hence, I suggest including a section that compares other vaccination experiences.
2. I think the Data and Methods section is underdeveloped, so the author should add more information about data collection and statistical analysis.
3. The Discussion section needs more information on policy implications that can help other regions.

#### Minor Comments

1. Some sentences need to be rewritten, and the text needs to be proofread.
